# Association of premarital pregnancy with adverse birth outcomes and its characteristics in Japan

**DOI:** 10.1186/s40834-025-00357-4

**Published:** 2025-04-09

**Authors:** Tasuku Okui, Naoki Nakashima

**Affiliations:** Medical Information Center, Kyushu University Hospital, Maidashi3-1-1 Higashi-Ku, Fukuoka City, Fukuoka prefecture 812-8582 Japan

**Keywords:** Japan, Premarital pregnancy, Preterm birth, Small-for-gestational-age, Low birth weight

## Abstract

**Background:**

A study investigating the association between premarital pregnancy and the adverse birth outcomes has not been conducted in Japan. This study aimed to investigate an association of premarital pregnancy with adverse birth outcomes and its characteristics in Japan, using national birth data.

**Methods:**

Birth data from the Vital Statistics: Occupational and Industrial Aspects for the fiscal years 2010, 2015, and 2020 were used. Firstborn and singleton births were used, and we restricted the data to infants born to Japanese parents. We defined the status of premarital pregnancy based on the length of marriage at the time of birth. Rates of preterm birth, term low birth weight (TLBW), and small-for-gestational-age (SGA) were used as outcomes. Log-binomial regression analysis was conducted to calculate the adjusted risk ratio of premarital pregnancy for each of the outcomes. Furthermore, logistic regression analysis was conducted to identify factors associated with premarital pregnancy.

**Results:**

Data from 888,459 births were included in the analysis. The results of log-binomial regression showed that the risk of premarital pregnancy was statistically significantly higher than that of postmarital pregnancy for all the outcomes, and the risk ratios were 1.65 (95% confidence intervals (CI):1.58, 1.72), 1.17 (95% CI:1.12, 1.22), and 1.12 (95% CI:1.08, 1.17) for preterm birth, TLBW, and SGA, respectively. The results of logistic regression analysis showed that lower non-manual workers, manual workers, and others were significantly and positively associated with premarital pregnancy compared to upper non-manual workers in terms of maternal and paternal occupations.

**Conclusions:**

It was shown that premarital pregnancy was associated with a higher risk of preterm birth, TLBW, and SGA and was positively associated with parental occupations such as lower non-manual workers and manual workers in Japan.

**Supplementary Information:**

The online version contains supplementary material available at 10.1186/s40834-025-00357-4.

## Background

Although childbearing is not always accompanied by marriage in many countries, the percentage of infants born to unmarried mothers is low in Japan, approximately 2% in recent years [1]. In contrast, premarital (bridal) pregnancy, where pregnancy occurs before marriage, is prevalent in Japan, and the proportion was approximately 20% among first-born births within marriage in recent years [2]. The proportion of premarital pregnancies increased from around 1980 until the early 2000s in Japan, but it has been showing a subtle decreasing trend since then [2].

Premarital pregnancy is known to be associated with adverse birth outcomes in some countries, and an association with preterm birth and low birthweight was observed [3, 4]. Preterm birth, low birthweight, and small-for-gestational-age (SGA) are major adverse birth outcomes and are risk factors for infant mortality [5, 6]. Moreover, they are risk factors for the occurrence of chronic diseases in adults [7, 8]. While studies investigating the association of premarital pregnancy with a mother’s workstyle and subsequent childbearing have been conducted in Japan [9, 10], a study investigating the association between premarital pregnancy and the adverse birth outcomes has not been conducted. In Japan, it is known that sociodemographic factors can affect adverse birth outcomes, and there is a possibility that premarital pregnancy is associated with adverse birth outcomes if premarital pregnancy is caused by sociodemographic reasons.

In Japan, it is known that the proportion of premarital pregnancy is high among teenagers and is positively associated with lower educational levels in Japan [2, 9], although the number of participants in the survey was relatively limited. Furthermore, studies investigating the characteristics of premarital pregnancy are scarce in Japan, and there have been no studies investigating the association between premarital pregnancy and sociodemographic characteristics of parents using national birth data (the Vital Statistics) in Japan.

In this study, we investigated the association between premarital pregnancy and adverse birth outcomes and the characteristics of premarital pregnancy in Japan using national birth data.

## Methods

### Data and data processing

In this study, we used birth data from the Vital Statistics: Occupational and Industrial Aspects for the fiscal years 2010, 2015, and 2020 in Japan. The data were provided by the Ministry of Health, Labour and Welfare on the basis of Article 33 of the Statistics Act. The Vital Statistics survey investigating occupations of parents is conducted every five fiscal years. In addition, the Vital Statistics survey is conducted based on birth certificates. Parents write basic information of infants and themselves in the birth certificates, and medical characteristics of infants are written by a medical professional. Specifically, we used data that included information on marital status, age, nationality, parity, and the occupation for each mother, as well as the sex, birth year, birth month, birth weight, gestational age (in weeks and days), and the number of fetuses for each infant. Furthermore, we used data on paternal occupation, household occupation, paternal nationality, and the starting year and month of cohabitation. The starting year and month of cohabitation represent the time of either the wedding or the beginning of cohabitation, whichever occurred first. Household occupation means the occupation (employment status) of the top earner of the household. Household occupation was classified into six types: households of farmers, self-employed workers, full-time workers at a smaller company, full-time workers at a larger company, others, and unemployed. “Full-time worker at a smaller company” referred to households where a full-time worker was employed in a company with fewer than 100 employees, while “full-time worker at a larger company” indicates households with a board member of a company or a full-time worker who does not fall under the “full-time worker at a smaller company” category.

Maternal age was grouped into 19 years or less, 20–24 years, 25–29 years, 30–34 years, 35–39 years, and ≥ 40 years. Parental occupations consist of 13 types, and we grouped them into occupational classes for ease of interpretation, as was conducted in previous studies [11–13]. They were classified into upper non-manual workers (administrative and managerial workers and professional and engineering workers), lower non-manual workers (clerical workers, sales workers, and service workers), manual workers (manufacturing process workers, transport and machine operating workers, construction and mining workers, and carrying, cleaning, packaging, and related workers), and others (unemployed persons, security workers, agriculture, forestry, and fishery workers, and workers in unclassifiable occupations).

We defined adverse birth outcomes as the occurrence of infants with preterm birth, term low birth weight (TLBW), or SGA. Preterm birth was defined as infants with a gestational age of less than 37 weeks, while the TLBW rate was defined as infants with a birth weight of less than 2500 g among infants with a gestational age of ≥ 37 weeks. SGA was defined based on the neonatal anthropometric chart in Japan. Infants with a birthweight below the 10th percentile on the chart, considering each combination of sex, parity, and gestational age (in weeks and days), were classified as SGA [14]. As the anthropometric chart covers only gestational weeks ranging from 22 to 41, we limited our analysis of SGA to birth data within this gestational period.

Premarital pregnancy was defined based on the length of marriage, which was calculated by subtracting the starting year and month of cohabitation from the birth year and month [2]. For instance, if the starting year and month of cohabitation were in April 2020 and the infant’s birthdate was in November 2020, the length of marriage would be 7 months (11 − 4 = 7). The Ministry of Health, Labour and Welfare in Japan proposed a method for defining premarital pregnancy by combining the length of marriage and gestational age [2], and premarital pregnancy is defined as a birth in which the length of marriage is shorter than the gestational age. Similarly, postmarital pregnancy can be defined as a birth in which the length of marriage is longer than the gestational age. However, because the length of marriage is defined in months (not days) in the data, many births are not classified as premarital or postmarital pregnancies and are not included in the analysis. As a result, births with longer gestational ages tend to be classified as premarital pregnancies among infants born with the same length of marriage, causing selection bias and making it challenging to investigate the association between premarital pregnancy and adverse birth outcomes. It is necessary to consider the definition in which the status of premarital and postmarital pregnancy of each birth is not influenced by the gestational age. Therefore, we defined premarital pregnancy as births with a length of marriage shorter than any gestational age in the data, while postmarital pregnancy was defined as births with a length of marriage longer than any gestational age in the data. The gestation weeks ranged from 17 to 50 weeks in the data. Hence, we defined premarital pregnancy as birth data with lengths of marriage shorter than 3 months and postmarital pregnancy as birth data with lengths of marriage longer than 12 months. A similar method for defining premarital pregnancy was used in a previous study [3].

### Statistical analysis

We narrowed the study population to first births of mothers and infants born within a marriage. Additionally, only singleton births were included in the analysis. Moreover, the relationship between marriage and childbirth varies depending on countries, and there are countries where childbirth does not accompany marriage. Therefore, we restricted the data to infants born to Japanese parents.

We tabulated the number of births by various birth characteristics based on the status of premarital pregnancy. Numbers and rates of preterm birth, TLBW, and SGA were also compared between premarital and postmarital pregnancy. Additionally, a log-binomial regression model was used to calculate risk ratio (RR), 95% confidence intervals (CI), and p-value for premarital pregnancy in relation to each birth outcome. In this analysis, maternal age group, household occupation, maternal occupation, paternal occupation, and the year of birth were used as explanatory variables. Moreover, logistic regression analysis was conducted to identify factors associated with premarital pregnancy, and odds ratio (OR), 95% CI, and p-value were calculated for each variable. The outcome variable in the logistic regression analysis was the status of premarital pregnancy, while the explanatory variables included maternal age group, household occupation, maternal occupation, paternal occupation, and the year of birth.

Complete-case analysis was conducted, and multiple imputation was used as a sensitivity analysis to address missing data in the regression analyses. For these analyses, two-sided tests were conducted, and a p-value less than 0.05 was considered statistically significant for the regression analyses. All analyses were conducted using R4.1.3 [15], with the utilization of packages such as lmtest [16] and mice [17]. The statistics shown in this study were created by the authors using data provided by the Ministry of Health, Labour and Welfare, and they are not statistics publicly released by the Ministry.

## Results

### Flowchart of study population

**Fig. 1:**
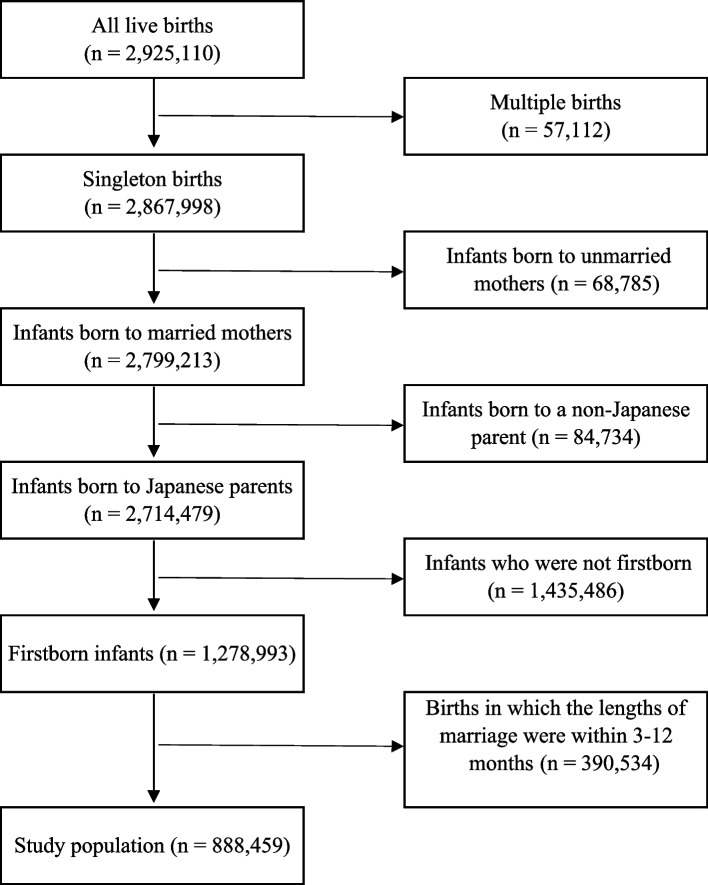
Flowchart of selecting birth data which were used in the analysis

Figure 1 shows flowchart of selection process for the births used in the analysis. The data for analysis comprised 888,459 births.

### Birth characteristics by the status of premarital pregnancy

Table 1 presents the number (%) of births categorized by various birth characteristics and the status of premarital pregnancy. Notably, the proportions of mothers aged ≤ 19 years and those aged 20–24 years among premarital pregnancies were 11.0% and 33.1%, respectively, while those among postmarital pregnancies were 0.2% and 4.8%, respectively. Similarly, the proportion of households with a full-time worker at a larger company among premarital pregnancies and postmarital pregnancies were 32.5% and 52.3%, respectively, and the proportions of the other households were higher among premarital pregnancies than among postmarital pregnancies. In addition, the proportions of upper non-manual workers among mothers and fathers for premarital pregnancies were 11.3% and 18.7%, respectively, while those for postmarital pregnancies were 19.3% and 32.8%, respectively.

**Table 1:** Number (%) of births by birth characteristics and the status of premarital pregnancy

Characteristics	Postmarital pregnancy	Premarital pregnancy
Total	850,536 (100.0)	37,923 (100.0)
Maternal age group		
19 years or less	1,366 (0.2)	4,185 (11.0)
20–24 years	40,694 (4.8)	12,559 (33.1)
25–29 years	282,127 (33.2)	10,038 (26.5)
30–34 years	325,554 (38.3)	6,419 (16.9)
35–39 years	161,713 (19.0)	3,718 (9.8)
40 years or more	39,082 (4.6)	1,004 (2.6)
Household occupation		
Farmer	7,983 (0.9)	600 (1.6)
Self-employed	48,361 (5.7)	3,083 (8.1)
Full-time worker at a smaller company	257,973 (30.3)	14,876 (39.2)
Full-time worker at a larger company	444,747 (52.3)	12,343 (32.5)
Other occupations	71,210 (8.4)	4,618 (12.2)
Unemployed	4,891 (0.6)	1,252 (3.3)
Missing	15,371 (1.8)	1,151 (3.0)
Maternal occupation		
Upper non-manual workers	164,661 (19.4)	4,291 (11.3)
Lower non-manual workers	228,236 (26.8)	8,143 (21.5)
Manual workers	16,744 (2.0)	904 (2.4)
Others	409,035 (48.1)	23,083 (60.9)
Missing	31,860 (3.7)	1,502 (4.0)
Paternal occupation		
Upper non-manual workers	278,740 (32.8)	7,098 (18.7)
Lower non-manual workers	300,700 (35.4)	12,675 (33.4)
Manual workers	167,354 (19.7)	12,055 (31.8)
Others	70,241 (8.3)	4,489 (11.8)
Missing	33,501 (3.9)	1,606 (4.2)
Sex		
Female	414,859 (48.8)	18,394 (48.5)
Male	435,677 (51.2)	19,529 (51.5)
Birth weight		
< 2500 g	75,605 (8.9)	4,374 (11.5)
2500–3999 g	769,282 (90.4)	33,321 (87.9)
4000 g or more	5,499 (0.6)	214 (0.6)
Missing	150 (0.0)	14 (0.0)
Gestational age		
36 weeks or less	38,097 (4.5)	2,597 (6.8)
37 weeks or more	812,303 (95.5)	35,309 (93.1)
Missing	136 (0.0)	17 (0.0)
Year		
2010	294,737 (34.7)	13,654 (36.0)
2015	300,031 (35.3)	13,909 (36.7)
2020	255,768 (30.1)	10,360 (27.3)

### Association between premarital pregnancy and adverse birth outcomes

Table 2 provides the numbers and rates of adverse birth outcomes categorized by the status of premarital pregnancy. The rates of preterm birth, TLBW, and SGA for infants born to mothers with premarital pregnancy were 6.8%, 6.9%, and 8.1%, respectively, while those for infants born to mothers with postmarital pregnancy were 4.5%, 6.1%, and 7.4%, respectively. Therefore, the rates of preterm birth, TLBW, and SGA for infants born to mothers with premarital pregnancy were higher than those for infants born to mothers with postmarital pregnancy.

**Table 2:** Number and rate of the adverse birth outcomes by the status of premarital pregnancy

Status of premarital pregnancy	Outcomes		
	Preterm birth	TLBW	SGA
Postmarital pregnancy	35,826 (4.5)	46,938 (6.1)	58,990 (7.4)
Premarital pregnancy	2,379 (6.8)	2,266 (6.9)	2,850 (8.1)

*TLBW* term low birthweight, *SGA* small-for-gestational-age

Table 3 presents the results of log-binomial regression analysis for each of the adverse birth outcomes. The RRs of premarital pregnancy were statistically significantly higher than post-marital pregnancy for all the outcomes, and RRs were 1.65 (95% CI:1.58, 1.72), 1.17 (95%CI:1.12, 1.22), and 1.12 (95%CI:1.08, 1.17) for preterm birth, TLBW, and SGA, respectively.

**Table 3:** The result of log-binomial regression analysis for each of the adverse birth outcomes

Outcomes and the status of premarital pregnancy	Adjusted RR (95% CI)^a^	*p*-value
Preterm birth		
Postmarital pregnancy	Reference	
Premarital pregnancy	1.65 (1.58, 1.72)	< 0.001
TLBW		
Postmarital pregnancy	Reference	
Premarital pregnancy	1.17 (1.12, 1.22)	< 0.001
SGA		
Postmarital pregnancy	Reference	
Premarital pregnancy	1.12 (1.08, 1.17)	< 0.001

*TLBW* term low birthweight, *SGA* small-for-gestational-age, *RR* risk ratio, *CI* confidence interval

^a^Maternal age group, household occupation, maternal occupation, paternal occupation, and birth year were adjusted

Supplementary Table 1 shows the results of log-binomial regression analysis using multiple imputation. The findings align with those from the complete-case analysis.

### Factors associated with premarital pregnancy

Table 4 displays the results of logistic regression analysis conducted to identify factors associated with premarital pregnancy. The maternal age group of 19 years or less showed a notably positive association with premarital pregnancy, and the OR was 117.36 (95%CI:109.38, 125.92). Regarding household occupation, the households other than the full-time worker at a larger company showed a significant and positive association with premarital pregnancy compared with the full-time worker at a larger company, and the odds ratio was the largest in unemployed household, with an OR of 3.97 (95%CI:3.64, 4.33). Regarding maternal and paternal occupations, individuals in the occupational classes other than upper non-manual workers were positively associated with premarital pregnancy compared to upper non-manual workers. Specifically, the ORs of lower non-manual workers, manual workers, and others were 1.11 (95%CI:1.07, 1.16), 1.09 (95%CI:1.01, 1.19), and 1.18 (95%CI:1.13, 1.22) for maternal occupation, respectively, and those for paternal occupation were 1.37 (95%CI:1.33, 1.41), 1.50 (95%CI:1.45, 1.55), and 1.45 (95%CI:1.39, 1.52), respectively.

**Table 4:** The result of logistic regression analysis of associated factors of premarital pregnancy

Characteristics	Adjusted OR (95% CI)	*p*-value
Maternal age group		
19 years or less	117.36 (109.38, 125.92)	< 0.001
20–24 years	13.02 (12.58, 13.47)	< 0.001
25–29 years	1.75 (1.70, 1.81)	< 0.001
30–34 years	Reference	
35–39 years	1.13 (1.08, 1.18)	< 0.001
40 years or more	1.25 (1.16, 1.34)	< 0.001
Household occupation		
Farmer	1.86 (1.69, 2.05)	< 0.001
Self-employed	1.94 (1.86, 2.03)	< 0.001
Full-time worker at a smaller company	1.52 (1.48, 1.56)	< 0.001
Full-time worker at a larger company	Reference	
Other occupations	1.71 (1.64, 1.77)	< 0.001
Unemployed	3.97 (3.64, 4.33)	< 0.001
Maternal occupation		
Upper non-manual workers	Reference	
Lower non-manual workers	1.11 (1.07, 1.16)	< 0.001
Manual workers	1.09 (1.01, 1.19)	0.026
Others	1.18 (1.13, 1.22)	< 0.001
Paternal occupation		
Upper non-manual workers	Reference	
Lower non-manual workers	1.37 (1.33, 1.41)	< 0.001
Manual workers	1.50 (1.45, 1.55)	< 0.001
Others	1.45 (1.39, 1.52)	< 0.001
Year		
2010	Reference	
2015	1.12 (1.09, 1.15)	< 0.001
2020	1.03 (1.00, 1.06)	0.053

*OR* odds ratio, *CI* confidence interval

Supplementary Table 2 presents the results of logistic regression analysis using multiple imputation. The findings align with those obtained from the complete-case analysis.

## Discussion

This study investigated an association of premarital pregnancy with adverse birth outcomes and its sociodemographic characteristics in Japan. As a result, premarital pregnancy was found to be associated with adverse birth outcomes, and some sociodemographic factors were found to be associated with premarital pregnancy. Here, we discuss possible reasons for this association, the implications of our findings, and limitations in this section.

Our findings indicated that premarital pregnancy carries a higher risk of adverse birth outcomes compared to postmarital pregnancy. To our knowledge, there is a paucity of studies that investigated associations between premarital pregnancy and adverse birth outcomes, and a study conducted in Korea also showed the associations of marriage preceded by pregnancy with preterm birth and low birth weight [3]. Unintended pregnancy and prenatal stress were pointed out as possible reasons for the association in the study [3]. A study conducted in Gibraltar also demonstrated that premarital conception is a risk factor for lower birth weight among teenage mothers [4]. As possible reasons for the association between premarital pregnancy and adverse birth outcomes in our study, sociodemographic characteristics of parents who experience premarital pregnancy and the existence of unmarried periods during pregnancy can be pointed out. Regarding sociodemographic characteristics of premarital pregnancy, premarital pregnancy is associated with lower socioeconomic status. In Japan, parental educational level and income level are known to impact adverse birth outcomes [18, 19]. Risk factors for preterm birth and low birth weight such as smoking and lower utilization of prenatal care are positively associated with lower socioeconomic status [20, 21]. Regarding the existence of unmarried periods for premarital pregnancy, unmarried status of mothers is known as a risk factor for adverse birth outcomes in Japan and other countries [22–24]. For instance, in Norway, single mothers exhibited lower dietary quality during pregnancy compared to married women [25]. It is also suggested that married individuals tend to take on more responsibility in terms of health-related activities, potentially influencing the health behaviors of pregnant women [24]. Therefore, there is a possibility that socioeconomic status and the existence of unmarried periods during pregnancy are reasons for the associations between premarital pregnancy and adverse birth outcomes.

Regarding factors associated with premarital pregnancy, the maternal age group of “19 years or less” showed a strong association with premarital pregnancy, which is consistent with previous findings [2]. The results suggest that a major factor of marriage for teenage women is pregnancy. In addition, parental and household occupations with lower socioeconomic status had a positive association with premarital pregnancy in this study, highlighting the persistence of socioeconomic disparities in recent years. One possible explanation is that premarital pregnancy is often associated with unplanned pregnancies, as evidenced by previous studies in Japan [26]. Lower socioeconomic status has been associated with unplanned pregnancies in Japan and other countries as well [27–29]. Additionally, contraceptive choices can be influenced by socioeconomic status [30, 31], and women with higher educational levels had a negative association with the use of unreliable or no contraception compared with those with lower educational level in Japan [32]. The year of birth was also significantly associated with premarital pregnancy; however, the reason for this finding is uncertain. The effects of factors such as contraception behaviors and the frequency of unplanned pregnancy are considered to differ depending on the year, and the effects of the year of birth might represent those of factors that were not taken into account in this analysis.

As an implication of this study, we have established that premarital pregnancy is a predictor for adverse birth outcomes in Japan. Consequently, it may be necessary to provide more support for women with premarital pregnancies. This support could include recommendation of use of antenatal care or advice on diet and lifestyle behaviors during antenatal care. In addition, an education for contraceptive use is also important to prevent unplanned pregnancy, particularly for men and women with lower socioeconomic status. Furthermore, it is important to investigate the association between premarital pregnancy and health status and behaviors in the future to gain a deeper understanding of the reasons behind the higher risk.

The strength of this study is that we used the Vital Statistics data, and the data cover all births in Japan. In contrast, there are some limitations in this study. First, we defined the length of marriage at the time of birth from the starting time of cohabitation, which represents the time of either the wedding or the beginning of cohabitation, whichever occurred first. Although the Ministry of Health, Labour and Welfare in Japan also uses this starting time of cohabitation to calculate length of marriage, it is considered that there are couples for whom the calculated length of marriage does not precisely correspond to the accurate length of marriage. Additionally, we defined premarital pregnancy as birth data with lengths of marriage shorter than 3 months for the methodological reason, while those birth data are a part of premarital pregnancy. Moreover, other unmeasured factors, such as maternal pregnancy conditions, comorbidities, the educational level, and smoking status were not available in the dataset. A study investigating these aspects would provide valuable insights into the reasons behind the results in this study. Furthermore, because this study targeted infant born to Japanese parents, the results may be generalizable only to Japan. It is meaningful to investigate the association also in other countries.

## Conclusions

This study examined the characteristics of premarital pregnancy and its association with adverse birth outcomes among infants born to Japanese parents in Japan. It was shown that the risks for preterm births, TLBW, and SGA were higher in premarital pregnancies than in postmarital pregnancies. In addition, premarital pregnancy was positively associated with parental occupations such as lower non-manual workers and manual workers.

## Supplementary Information

Additional file 1.

## Data Availability

The data that support the findings of this study are available from the Ministry of Health, Labour and Welfare in Japan but restrictions apply to the availability of these data, which were used under license for the current study and are not publicly available. Data are however available from the Ministry of Health, Labour and Welfare if the Ministry permits use of the data.

## Abbreviations

CI: Confidence intervals
OR: Odds ratio
RR: Risk ratio
SGA: Small-for-gestational-age
TLBW: Term low birthweight
