# Supplementary material for: Association of premarital pregnancy with adverse birth outcomes and its characteristics in Japan
Source: Contracept Reprod Med. 2025 Apr 9;10:30. doi: 10.1186/s40834-025-00357-4 (PMC11980210; doi:10.1186/s40834-025-00357-4)
Supplement: Supplementary file 1 — Additional file 1. [file 40834_2025_357_MOESM1_ESM.pdf]

Supplementary Table 1. The result of log-binomial regression analysis using multiple imputation.

Outcomes and the status of premarital pregnancy	Adjusted RR (95% CI)*	p-value
Preterm birth		
Postmarital pregnancy	Reference	
Premarital pregnancy	1.67 (1.60, 1.74)	<0.001
TLBW		
Postmarital pregnancy	Reference	
Premarital pregnancy	1.18 (1.13, 1.23)	<0.001
SGA		
Postmarital pregnancy	Reference	
Premarital pregnancy	1.12 (1.08, 1.16)	<0.001

TLBW, term low birthweight; SGA, small-for-gestational-age; RR, risk ratio; CI, confidence interval

*Maternal age group, household occupation, maternal occupation, paternal occupation, and birth year were adjusted.

Supplementary Table 2. The result of logistic regression analysis using multiple imputation.

Characteristics	Adjusted OR (95% CI)	p-value
Maternal age group		
19 years or less	115.41 (107.81, 123.54)	<0.001
20–24 years	13.10 (12.68, 13.54)	<0.001
25–29 years	1.77 (1.71, 1.83)	<0.001
30–34 years	Reference	
35–39 years	1.14 (1.10, 1.19)	<0.001
40 years or more	1.26 (1.18, 1.35)	<0.001
Household occupation		
Farmer	1.88 (1.70, 2.08)	<0.001
Self-employed	1.93 (1.84, 2.03)	<0.001
Full-time worker at a smaller company	1.51 (1.47, 1.56)	<0.001
Full-time worker at a larger company	Reference	
Other occupations	1.70 (1.63, 1.77)	<0.001
Unemployed	3.90 (3.51, 4.33)	<0.001
Maternal occupation		
Upper non-manual workers	Reference	
Lower non-manual workers	1.12 (1.07, 1.16)	<0.001
Manual workers	1.10 (1.01, 1.20)	0.027
Others	1.18 (1.14, 1.23)	<0.001
Paternal occupation		
Upper non-manual workers	Reference	
Lower non-manual workers	1.36 (1.32, 1.41)	<0.001
Manual workers	1.50 (1.44, 1.55)	<0.001
Others	1.46 (1.39, 1.53)	<0.001
Year		
2010	Reference	
2015	1.12 (1.09, 1.15)	<0.001
2020	1.03 (1.00, 1.06)	0.051

OR, odds ratio; CI, confidence interval
